# Expression of Matrix Metalloproteinase-9 and Tissue Inhibitor of Metalloproteinase-1 in Endometriosis Menstrual Blood

**DOI:** 10.3390/diagnostics10060364

**Published:** 2020-06-02

**Authors:** Tita Husnitawati Madjid, Dennis Fachmi Ardiansyah, Wiryawan Permadi, Bethy Hernowo

**Affiliations:** 1 Department of Obstetrics and Gynecology, Hasan Sadikin General Hospital—Universitas Padjadjaran, Bandung 40161, Indonesia; denzfa80@gmail.com (D.F.A.); wiryawanpermadi@yahoo.com (W.P.); 2 Department of Pathology, Hasan Sadikin General Hospital—Universitas Padjadjaran, Bandung 40161, Indonesia; bethy_hernowo@yahoo.com

**Keywords:** MMP-9, TIMP-1, endometriosis

## Abstract

Endometriosis is a gynecological disease characterized by the presence of endometrial-like tissue outside the uterine cavity. Remodeling of the extracellular matrix (ECM) is a prerequisite for tissue implantation. The presence of matrix metalloproteinase (MMP) and its inhibitor, tissue inhibitor of metalloproteinase-1 (TIMP-1), in shed endometrium cells has a significant role in ECM degradation. A case–control study was performed to find other diagnostic markers using menstrual blood. We examined a sample of 68 women who visited the gynecology clinic in Dr Hasan Sadikin General Hospital, 40% of whom were confirmed to have endometriosis, and the rest tested negative by histopathological examination. All endometriotic cases presented MMP-9 and TIMP-1 expression with different cell distribution. MMP-9 expression in endometriosis patients was increased compared to the controls (*p* = 0.002). Expression of MMP-9 in >80% of endometrial cells was associated with a higher risk for endometriosis (OR 4.44 95% CI 1.31 to 15.56) compared to MMP-9 expression in 50%–80% of cells. TIMP-1 cell expression in women with endometriosis was lower than in the control group (*p* = 0.030). Subjects with TIMP-1 expression in 20%–50% of endometrial cells had a higher risk for endometriosis (OR 4.5, 95%CI 1.21–17.42) compared those with TIMP-1 expression in 50%–80% of cells. These expressions levels can be useful to predict endometriosis.

## 1. Introduction

Endometriosis is a common but poorly understood gynecological disorder that affects 2%–10% of reproductive-age women [1]. Endometriosis features the implantation of endometrial tissue outside the uterine cavity and is associated with chronic pelvic pain and infertility [1,2]. Approximately 176 million women are predicted to suffer from endometriosis [2].

Despite its prevalence and impact on fertility, the pathophysiology of endometriosis is yet to be elucidated. In 1927, Sampson proposed that the regurgitation of menstrual blood from the uterine cavity through the fallopian tube is responsible for its implantation outside the uterine cavity [3]. Menstrual regurgitation is estimated to occur in 76% to 90% of women, a percentage much higher than the expected rate of endometriosis. This indicates the possible involvement of other factors that promote endometrial cell implantation. 

Degradation of extracellular matrix (ECM) tissue by proteolytic enzymes is thought to be involved in the process of endometrial cell implantation. Among these enzymes, fibronectin, laminin, and particularly matrix metalloproteinase (MMP) could play a role in endometrial cell implantation [4]. 

Tissue inhibitor of metalloproteinase (TIMP) is a protein that inhibits metalloproteinase enzymes. These proteins include TIMP-1, TIMP-2, TIMP-3, and TIMP-4. TIMP-1 inhibits the active forms of MMP-2 and MMP-9. The balance of MMP and TIMP plays a crucial role in physiological processes such as tissue repair, embryogenesis, trophoblast invasion, and the process of menstruation, as well as in pathological processes such as arthritis and the invasion of tumor cells, although pathological processes are also influenced by other factors such as genetics, immunity, and other unknown factors [4,5,6].

A definite diagnosis of endometriosis still relies on invasive procedures such as laparoscopy, which is used to identify, search, and observe lesions and is followed by removal and analysis of lesion biopsies; approximately 54%–67% of suspected lesions are histologically confirmed as endometriosis [7]. Laparoscopy is useful when lesions or other anatomical abnormalities occur, but this is not always the case. As a result, delays in endometriosis diagnosis frequently occur, which leads to difficulties in managing this condition. 

Investigations by ultrasound, computerized tomography scanning, magnetic resonance imaging, and carcinoantigen-125 (CA-125) evaluation are noninvasive diagnostic means but are mainly useful for diagnosing severe conditions [7,8]. Early disease identification is crucial in preventing the progression of the disease and facilitating its treatment. 

It may be possible to conduct a noninvasive diagnostic examination to detect endometriosis prior to the manifestation of anatomical abnormalities by way of detecting living endometrial cell proteins in menstrual blood. These proteins are similar to those found in endometriosis cells. Of the various types of proteins found in endometriosis cells, MMP-9 and TIMP-1 were detected in peritoneal fluid and menstrual blood [5]. 

Given that these proteins play an important role in the early pathophysiology of the disease before anatomical abnormalities occur, we hypothesized that MMP-9 and TIMP-1 in endometrial cells isolated from menstrual blood can be used as markers to support the diagnosis of endometriosis. 

## 2. Methods

### 2.1. Study Design and Subject Selection

This study was approved by the Committee for Medical Research, Universitas Padjadjaran, Indonesia (122/FKUP-RSHS/KEPK/Kep/EC/2009, 11 September 2009). We conducted a case–control study of patients with endometriosis who attended the gynecology clinic of Dr Hasan Sadikin General Hospital, Bandung, Indonesia, from June to September 2010. Cases and controls were chosen based on inclusion and exclusion criteria. This study was approved by the Committee for Medical Research, Universitas Padjadjaran, Indonesia (122/FKUP-RSHS/KEPK/Kep/EC/2009, 11 September 2009). 

### 2.2. Inclusion and Exclusion Criteria

The inclusion criteria were as follows: cases were women who attended the clinic and received a diagnosis of endometriosis stages III and IV through anamnesis, physical examination, ultrasound and had the diagnosis confirmed through histopathological examination following diagnostic laparoscopy. Controls were selected from women who attended the clinic with pelvic pain not associated with endometriosis following anamnesis, physical examination, ultrasonography, and confirmed through histopathological results. The exclusion criteria were women with confirmed endometriosis who had previously received estrogen and/or progesterone therapy, along with women who were diagnosed with pelvic infection and/or malignancy. Subjects had to give verbal and written consent before they were enrolled in the study. 

### 2.3. Sample Size Calculation

A priori power calculation was not conducted due to the lack of standard deviation value in the population. An initial number of 30 subjects per group was chosen, and interim analysis was selected as an alternative to determine the standard deviation. Sixty-eight subjects (*n* cases = 30, *n* controls = 38) were enrolled, and subsequent power analysis revealed the value of 1-β = 27.5%.

### 2.4. MMP-9 and TIMP-1 Assessment

MMP-9 and TIMP-1 were assessed from menstrual blood through immunocytochemistry. To assess MMP-9 and TIMP-1 in menstrual blood, 1 mL (20 drops) of menstrual blood was collected during the 2nd or 3rd day of the menstrual cycle and diluted into 20 ml of preservative. Samples were incubated at room temperature for 30 min and centrifuged at 744× *g* for 15 min. The precipitate was diluted and transferred to a glass slide. Following antigen retrieval, sections were incubated in 0.3% H_2_O_2_ (30% stock H_2_O_2_ diluted in methanol) and washed in PBS for 2 × 5 s. Sections were incubated in blocking reagent for 2 × 5 minutes, after which the blocking reagent was tipped off the slide, and a primary antibody (anti-MMP9 antibody, 1:1000, ab38898, Abcam; recombinant anti-TIMP-1 antibody, 1:1000, ab211926, Abcam) was applied. Sections were incubated in primary antibody for 1 hour, washed in PBS for 3 times, 5 s each time, and incubated in secondary antibody (R.T.U Biotinylated Universal Secondary Antibody BP-1400, Vector), for 2 times, 5 s each time. Color was developed using chromogen 3,3′-diaminobenzidine (DAB), and the slides were counterstained using Mayer’s hematoxylin (Sigma). Sections were dehydrated in 70%, 80%, and 90% industrial methylated spirit (IMS) for 3 min each, and mounted in xylol for 3 min.

### 2.5. Statistical Analysis

Subject characteristics were analyzed through descriptive statistics. The chi-squared test was used to examine the relationship between MMP-9, TIMP-1, and endometriosis; correlation was analyzed using Spearman’s rank correlation coefficient. A *p* value < 0.05 was considered significant. Statistical analysis was performed using SPSS version 13.0 (IBM Corp.).

## 3. Results

Sixty-eight subjects were enrolled from June to September 2010 (*n* cases = 30, controls = 38). The mean (SD) age of the subjects with endometriosis was 34 (6.8), and that of the controls was 31.6 (8.6); the mean body mass index (BMI) of the cases was 23 (3.5), and that of the controls was 22.8 (3.9). There was no statistically significant difference in the characteristics of the cases and the controls (Table 1).

**Figure 1:**
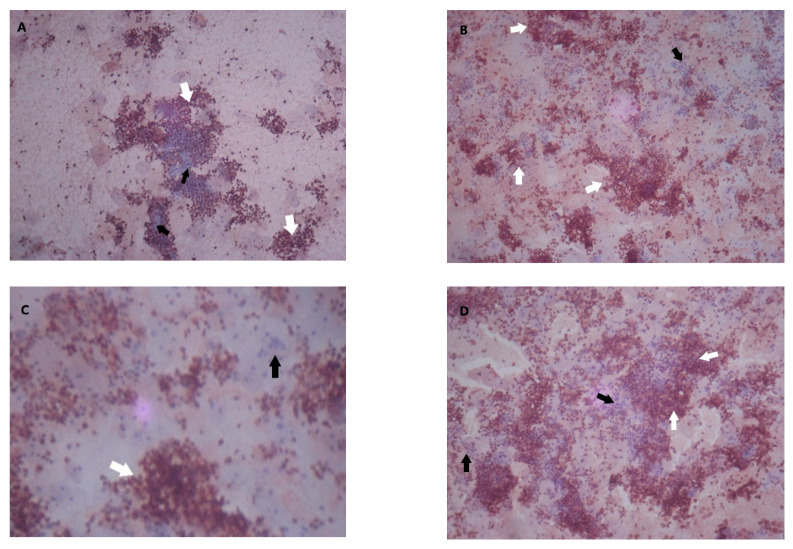
Matrix metalloproteinase (MMP)-9 and tissue inhibitor of metalloproteinase (TIMP)-1 staining in endometrial epithelial tissue. White arrows, positive staining; black arrow, negative staining. (**A**) MMP-9 weak staining (20%–50% positive staining per field of view); (**B**) MMP-9 moderate staining (50%–80% positive staining per field of view); (**C**) MMP-9 strong staining (>80% positive staining per field of view); (**D**) TIMP-1 moderate staining (50%–80% positive staining per field of view).

The intensity of MMP-9 and TIMP-1 staining in endometrial cells was categorized on the basis of the percentage of positive staining per field of view from weak to moderate and strong (Figure 1). Strong MMP-9 staining was found in 66% of endometriosis cells, compared to 26.3% of endometrial cells in the controls (OR 4.44, 95%CI 1.31–15.26, *p* < 0.005) (Table 2).

MMP-9 and TIMP-1 staining patterns were negatively correlated (Rs = −0.613, *p* < 0.001) in normal women (Table 3). On the other hand, there was no inverse correlation between MMP-9 and TIMP-1 staining in women with endometriosis (Rs = −0.196, *p* = 0.30).

## 4. Discussion

Matrix metalloproteinases are a family of proteolytic enzymes with the capability to degrade ECM components, whose role in the process of menstruation has been extensively studied [9,10,11,12]. MMP-1–3 and MMP-7–9 were demonstrated to be expressed in endometrial epithelium, stroma, and immune cells throughout the menstrual cycle, more strongly during the menstrual phase compared to the proliferative and stromal phases [10,12]. MMP-9 or gelatinase B can degrade collagens and is thought to contribute to degrading the basement membrane of the endometrium during the menstrual phase [12]. However, other functions of MMP-9 include angiogenesis, which was proposed to influence the growth of ectopic endometrial tissue.

MMPs are regulated by TIMP-1–4, with each substrate possessing its own activity against specific MMPs. TIMP-1 acts against membrane-type MMPs (MT1-, MT3-, MT-5MMP) and pro-MMP9, whereas TIMP2–4 has a wider range of activity [10]. 

MMP-9 and MMP-2 were localized in the endometrial tissue by Zhang (2002) in a significantly greater amount during the menstrual phase compared to other phases of the menstrual cycle [9]. In the uterine endometrial tissue of women with endometriosis, Collette and Maheux (2006) showed a heightened expression of MMP-9 through zymography and ELISA, but their samples were taken during the proliferative phase of the menstrual cycle [13]. During the menstrual phase, the examination of MMP expression in menstrual serum was conducted by Malik (2006), who investigated MMP-2 and MMP-9 by zymography and showed that the expression of the latent and the active forms of MMP-9 were similar in women with endometriosis and in those without [11]. Collette et al. followed their previous finding on MMP-9 by examining the expression of TIMP-1 and the MMP-9/TIMP-1 protein and mRNA ratios, but they did not find a statistically significant difference in TIMP-1 expression between women with and without endometriosis [13].

To our knowledge, this is the first study examining the expression of MMP-9 and TIMP-1 in menstruated endometrial cells. This study showed that MMP-9 is expressed in menstruated endometrial epithelial cells in women with and without endometriosis. Furthermore, it is more strongly expressed in women with endometriosis (*p* = 0.002; Table 2). The odds ratio of endometriosis is increased by the stronger staining of MMP-9 (OR 4.44, 95% CI 1.31–156).

In the current study, MMP-9 and TIMP-1 appeared to be negatively correlated (rs= –0.613, *p* < 0.001) in the controls. By contrast, this result was not found in endometriosis patients, for whom a negative, though statistically insignificant, correlation was found (rs = –0.196, *p* = 0.30). 

To date, the mechanisms of endometriosis development are still under investigation. Retrograde menstruation occurs in 76% to 90% of women. Meanwhile, the prevalence of endometriosis is much lower, about 6.2% to 8.2%, which indicates the involvement of other factors in determining the susceptibility to endometriosis. The latest research has indicated the involvement of genetic, immunological, and hormonal factors. There is a marked overexpression of estrogen receptor ERβ in the endometriotic tissue, which contributes to the suppression of progesterone activity in eutopic endometriosis compared to normal tissue [14]. Progesterone is a known regulator of MMP activity, and a reduction of progesterone receptor expression has been shown to be associated with MMP-9 overexpression [14,15].

Another possible consequence of the overexpression of estrogen receptor in the endometriotic tissue is its impact on certain signaling pathways responsible for cell cycle progression and cellular survival. Lagana et al. (2017) hypothesized that a deregulation of the Wnt/β-catenin pathway may result in a cascade of events that eventually provides an optimum environment for the development of endometriosis [16]. Indeed, in a study using human endometrial stromal cell (HESCs) gathered from eutopic endometriosis tissue, Xiong et al. (2015) discovered that the β-catenin signaling pathway was activated following estradiol (E_2)_ stimulation; the same stimulation also induced the production of vascular endothelial growth factor (VEGF) and MMP-9, whereas treatment using β-catenin siRNA reversed this effect [17]. This result is further supported by Zhang et al (2016), who demonstrated an increased amount of MMP-9 in the endometrial tissue of women with endometriosis, as well as a dose-dependent increase of MMP-9 and β-catenin expression in response to E_2_ stimulation [18]. While there is still a need to further elucidate the interaction between these factors, our findings support an increased expression of MMP-9 in endometriosis. [13,19]. In our study, there was an increase in MMP-9 expression and a decrease in TIMP-1 expression in endometrial cells isolated from the menstrual blood of patients with endometriosis. This study proved that the examination of MMP-9 and TIMP-1 expression in endometrial menstrual blood cells may support the diagnosis of endometriosis. This menstrual blood test is not invasive or expensive and is relatively easy compared to other existing diagnostic measures.

As preliminary work, this study is still limited in many ways. First, our subjects consisted of only Stage 3 and 4 endometriosis patients. To confirm the effectiveness of the menstrual blood test in detecting early endometriosis, a larger study with samples consisting of Stage 1 and 2 endometriosis patients is required. Second, a comparison between the effectiveness of this test and that of laparoscopy to detect endometriosis is required, as well as sensitivity and specificity analyses. 

## 5. Conclusions

This study showed that the expression of MMP-9 is directly related to the presence of endometriosis, while TIMP-1 expression is inversely related to endometriosis. As such, these proteins have the potential to be developed into a cheap and noninvasive diagnostic measure for endometriosis. 

## Figures and Tables

**Table 1:** Research subject characteristics. BMI, body-mass index.

	Characteristics	Group		*p*-Value
		Endometriosis (*n* = 30)	Control (*n* = 38)	
1.	Age (years)			*t* = 1.225 *p* = 0.225
	X (SD)	34.0 (6.8)	31.6 (8.6)	
	Median	35	31.0	
	Distance	20–49	17–54	
2.	Occupation			x^2^ = 2.653 *p* = 0.618
	Housewife	18	24	
	Working	12	14	
3.	Marital Status			x^2^ = 0.161 *p* = 0.688
	Married	27	33	
	Unmarried	3	5	
4.	Social economic			x^2^ = 4.565 *p* = 0.102
	a. High	0	5	
	b. Med	12	11	
	c. Low	18	22	
5.	BMI (kgs/m^−2^)			*t* = 0.230 *p* = 0.819
	X(SD)	23.0 (3.5)	22.8 (3.9)	
	Range	17.2–30.3	15–29	

**Table 2:** MMP-9 and TIMP-1 expression in endometrial menstrual blood cells in endometriosis patients and controls.

Variables	Group				OR (95% CI)	*p*-Value
	Endometriosis		Control			
	n	%	n	%		
1. MMP-9						0.002
20–50	1	3.3	8	21.1	0.28 (0.01–2.89)	
50–80	9	30	20	52.6	1.0	
>80	20	66.7	10	26.3	4.44 (1.31–15.56)	
2. TIMP-1						0.030
<20	5	16.7	5	13.2	3.0 (0.53–17.73)	
20–50	15	30	10	26.3	4.5 (1.21–17.42)	
50–80	7	23.3	21	55.2	1.0	
>80	3	10	2	5.3	1.5 (0.46–50.90)	

**Table 3:** Correlation between MMP-9 and TIMP-1 distributions.

MMP-9 Distribution	TIMP-1 Distribution				*p*-Value
	<20%	20%–50%	50%–80%	>80%	
1. Endometriosis					x^2^ = 3.981
20%–50%	0	1	0	0	*p* = 0.675
50%–80%	0	5	3	1	r_s_ = −0.196
>80%	5	9	4	2	*p* = 0.30
2. Normal					x^2^ = 25.053
20%–50%	1	0	5	2	*p* =< 0.001
50%–80%	0	5	15	0	r_s_ = −0.613
>80%	4	5	1	0	*p* =< 0.001

r_s_, Spearman’s rank correlation coefficient.
